# Preclinical Safety of the Root Extract of *Polygala tenuifolia* Willdenow in Sprague-Dawley Rats and Beagle Dogs

**DOI:** 10.1155/2014/570134

**Published:** 2014-11-05

**Authors:** Ki Young Shin, Beom Young Won, Hyun Jee Ha, Yeo Sang Yun, Hyung Gun Lee

**Affiliations:** ^1^Research & Development Center, Braintropia Co. Ltd., Anyang-si, Gyeonggi-do 431-716, Republic of Korea; ^2^Department of Microbiology, College of Natural Science, Dankook University, Cheonan-si, Chungnam 330-714, Republic of Korea

## Abstract

The root of *Polygala tenuifolia* Willdenow has been used for the treatment of insomnia, depression, and amnesia. However, the toxicological properties of the herb have been overlooked, because it has been used for a long time for various purposes. In this study, we evaluated the preclinical safety of the root extract in rats and beagle dogs. First, the acute oral toxicity was tested in both rats and dogs. In the rats, only one female of 2 g/kg died, but no treatment-related death or clinical and gross findings were observed after the administration. No toxicological changes or mortalities related to the test substance were also observed after the administration in the dogs. Although vomiting, discoloration, or hemorrhage was found in some dogs, there were no serious abnormalities. Second, the subchronic toxicity was investigated in the rats. Two animals were found dead in the female group of 1,000 mg/kg/day, but there were no abnormal findings associated with the test substance. There also were no adverse effects on the clinical signs, body weight, and hematological and biochemical findings. Therefore, our results showed that the acute or subchronic toxicity of the root extract of *Polygala tenuifolia* might not be toxic to rats and dogs.

## 1. Introduction

Traditional oriental herbal prescriptions have regained popularity over the past decade. They are used widely for the treatment and prevention of various diseases [1, 2], because their long history of clinical application and natural origin seem to guarantee that the prescriptions will be effective and nontoxic [3]. Each plant used in such herbal prescriptions can provide opportunities for the development of herbal food products, dietary supplements, and functional foods [4]. However, the toxicological aspects of medicinal herbs or naturally occurring functional foods have been neglected due to their long history of use. It has been demonstrated that medicinal plants may have undesirable properties [5], because some individuals taking herbal medicines have reported certain side effects. Therefore, the use of any plant for medicinal purposes by no means guarantees the safety of such a plant. This fact raises concerns about the potential toxic effects resulting from short-term and long-term use of such medicinal plants. Data from acute and subchronic toxicity studies on medicinal plants or naturally occurring functional foods should be obtained to increase confidence in the safety of their use, particularly in the development of pharmaceuticals [6].* Polygala tenuifolia* Willdenow is a perennial herbaceous plant distributed widely in China and Korea. In Asia, it is a well-known traditional medicine for the treatment of phlegm and detumescence. Traditional medicines are prepared from the roots of* P. tenuifolia* because of its expectorant, tonic, tranquilizing, and antipsychotic properties [7–9]. In particular, the herb is used against insomnia, neurasthenia, amnesia, depression, anxiety-related palpitations, restlessness, disorientation, dementia, and memory failure [10, 11]. Previously, we reported that the root extract of* P. tenuifolia* was shown to enhance memory and cognitive function in two animal models and two human models [12–15]. The extract has been shown to contain C-glycosides, triterpene saponins, sucrose esters, and oligosaccharide esters [16, 17]. It also contains various substances, such as tenuigenin, tenuifolin, DISS (3,6′-disinapoyl sucrose), and TMCA (3,4,5-trimethoxycinnamic acid), which have been shown to have proliferative and protective effects on hippocampal neurons [18, 19]. Several researchers have demonstrated the effects of* P. tenuifolia*, but information on its safety is lacking. Therefore, systematic evaluation of the safety of the root extract of this herb is necessary for the development of new foods or drugs. In this study, an alcohol extract from the dried root of* P. tenuifolia* was prepared, and its safety was evaluated using an acute oral toxicity test and a subchronic oral toxicity test in Sprague-Dawley rats and beagle dogs in accordance with two standards: the Korea Food and Drug Administration Notification numbers 2005-60 and 2005-79.

## 2. Materials and Methods

### 2.1. Preparation of the Root Extract of* Polygala tenuifolia*

The dried root extract of* Polygala tenuifolia* (500 g) was refluxed with 75% ethanol for 4 hr in a boiling water bath. This procedure was repeated twice and the ethanol solution was concentrated under a vacuum. The resulting concentrated ethanol fraction (125 g) of the plant root was used for this study [12].

### 2.2. Experimental Animals and Animal Husbandry

Six-week-old specific pathogen-free Sprague-Dawley (HSD; SD) rats were used. Six animals of each sex for the acute oral toxicity study and 40 animals of each sex for the subchronic oral toxicity study were received from Koatech Co. (Pyeongtaek-si, Gyeonggi-do, Korea), Ltd. For the acute oral toxicity study, six beagle dogs of each sex at 6 months of age were obtained fromtheHanlim Laboratory Animals Company (Hwaseong-si, Gyeonggi-do, Korea). The animal room was maintained at a temperature of 23 ± 3°C, with a relative humidity of 55 ± 15%, air ventilation of 10–20 times/h, and ambient light (150–300 Lux) controlled to produce a 12 h light/dark cycle. Animals were given irradiation-sterilized pellet feed and ground water disinfected by an ultraviolet sterilizer and ultrafiltered ad libitum. The dogs were reared in stainless steel cages (8000 × 900 × 750 mm), and identification cards showing their study number and animal number were attached to the cages.

### 2.3. Study Design Overview

This study was conducted in compliance with the Good Laboratory Practice (GLP) and Test Guidelines of the Organization for Economic Cooperation and Development [20, 21] and the Korea Food and Drug Administration [22, 23] at the GLP Institute approved by the KFDA (Korea Food and Drug Administration). The study protocol was approved by the Institutional Animal Care and Use Committee of the institute, which is accredited by the Association for Assessment and Accreditation of Laboratory Animal Care International, 2010.

#### 2.3.1. Acute Oral Toxicity Study in Rats

After a 7-day quarantine and acclimatization period, ten healthy animals of each gender at 6 weeks of age were used. The study included two groups: a control group given no treatment and an experimental group given 2,000 mg/kg, based on a preliminary study (data not shown). Each group consisted of five rats of each sex, and body weight ranges at dosing were 161.61–168.38 g for males and 144.98–158.17 g for females. The test substance was administered orally to each group. Thereafter, clinical signs and body weight were observed throughout the 15-day experimental period, and gross findings were observed on the scheduled necropsy day. This study was carried out in accordance with the standard operating procedures of SNUH-GLP (Non-Clinical Research Center, Biomedical Research Institute, Seoul National University Hospital).

#### 2.3.2. Acute Oral Toxicity Study in Beagle Dogs

Six male and six female dogs were divided into three groups of four animals; then, two dogs of each gender were allocated. The study included three groups: a control group given no treatment and experimental groups exposed to 1,000 mg/kg and 2,000 mg/kg. The body weight ranges at dosing were 7.269–8.785 kg for males and 6.531–7.449 kg for females. The test substance was administered orally to each group. Thereafter, clinical signs, mortality, and body weight were observed throughout the 15-day experimental period, and gross findings were observed on the scheduled necropsy day. This study was carried out in accordance with the standard operating procedures of Preclinical Research Center, ChemOn (Yongin, Gyeonggi-do, Korea) Inc.

#### 2.3.3. Subchronic Oral Toxicity Study in Rats

After a 7-day quarantine and acclimatization period, forty healthy animals of each gender at 6 weeks of age were used. A dose of 1,000 mg/kg/day was set as the high dose considering the results of a preliminary 1-week subchronic oral toxicity study (data not shown) and the characteristics of the test substance. Doses of 600 and 360 mg/kg/day were set as the middle and low doses, respectively, using a common ratio of 1.67. The vehicle-treated group was administrated with sterile water for injection only. Each group consisted of 10 rats of each sex, and 10 rats of each sex and group were killed after a 90-day treatment period. Body weight ranges at the beginning of dosing were 169.56–190.56 g for males and 125.30–142.00 g for females. The test substance was administered repeatedly by the oral route to each group for 90 days. The following items were examined during the experimental period: clinical signs, body weight, food and water consumption, ophthalmic examination, urinalysis, hematology, blood clotting time, serum biochemistry, necropsy findings, organ weight, and histopathology. This study was carried out in accordance with the standard operating procedures of Preclinical Research Center, ChemOn Inc.

### 2.4. Observation and Examination Items of the Acute Oral Toxicity Study in Rats

#### 2.4.1. Clinical Signs

In the rat studies, clinical signs and mortality were observed continuously for the first 1 h after administration and every hour for 6 h. Each animal was observed daily throughout the entire 15-day experimental period. Abnormal type and severity of signs, as well as the observation day and time, were recorded. The day of administration was set as day 1.

#### 2.4.2. Body Weight

Each rat was measured on days 1, 3, 7, and 14 using an electronic balance (Sartorius Co., Gottingen, Lower Saxony, Germany).

#### 2.4.3. Necropsy

On the scheduled necropsy day (day 15), all surviving animals were euthanized by exsanguination from the abdominal aorta and abdominal vena cava under a CO_2_ gas overdose and examined grossly for abnormalities of the body surface and internal organs.

### 2.5. Observation and Examination Items of the Acute Oral Toxicity Study in Dogs

#### 2.5.1. Clinical Signs

In the dog studies, the clinical signs and mortalities were checked every hour for 6 hours after administration. Each animal was observed daily throughout the entire 15-day experimental period. Abnormal type and severity of signs, as well as the observation day and time, were recorded. The day of administration was set as day 1.

#### 2.5.2. Body Weight

All dogs were individually weighed before administration and on days 1, 3, 7, and 14 after the administration.

#### 2.5.3. Necropsy

In the dog studies, the animals were anesthetized with ketamine (Yuhan Yanghaeng) and rompun (Bayer Korea) and euthanized by exsanguinations from the axillary artery and vein. All organs in the thoracic and abdominal cavities were observed grossly and the gross findings were recorded.

### 2.6. Observation and Examination Items of the Subchronic Oral Toxicity Study in Rats

#### 2.6.1. Clinical Signs

All animals were observed daily for clinical signs and mortality. The type, date of occurrence, and the severity of the signs were recorded individually. The initial day of administration was set as day 1.

#### 2.6.2. Body Weight

Animals were weighed on the initial day of administration, once per week during the experimental period, and on the day of the necropsy. The body weight at the necropsy was measured after an overnight fast.

#### 2.6.3. Food and Water Consumption

Food and water consumption were measured on the initial day of administration and then once per week during the experimental period. The amounts of food and water were measured before they were supplied to each cage, and the food and water remaining the next day were measured to calculate the difference, which was regarded as daily food and water consumption (g/rat/day).

#### 2.6.4. Ophthalmic Examination

In the last week of observation, a mydriatic (Ocuhomapin, Lot number 013118, Samil Pharm Co., Korea) was dropped into both eyes of each animal to facilitate mydriasis after observing the external appearance of the eyes of five males and five females per group. The anterior parts of the eyes, the optic media, and the ocular fundus were then observed with an ophthalmoscope and a fundus camera (Genesis, Kowa Co., Tokyo, Japan). No abnormal signs were observed during the ophthalmic examination. Therefore, no eye photographs or further examinations were performed.

#### 2.6.5. Urinalysis

Five males and five females per group were housed in metabolic cages for urine collection in the last week of observation, and fresh urine samples (about 1 mL) were collected for 3 h and used for urinalysis and urine sediment tests. Additionally, the total urine volume was measured from urine samples collected for 24 h. Test strips (Multistix 10 SG, Siemens, Washington, DC, USA) were dipped in about 0.3 mL of urine and the specific gravity, pH, protein, glucose, ketone bodies, occult blood, bilirubin, urobilinogen, and nitrite levels were analyzed with an automatic analyzer (CliniTek 100, Siemens, Ames Division, Miles Laboratory, USA). Urine color was observed with the naked eye, and the result was input into the automatic analyzer. Approximately 0.7 mL of urine was centrifuged (Hanil MF300, Seoul, Korea) for 5 min. The sediment was stained using the Sternheimer-Malbin method (Sternheimer and Malbin, 1951) and red blood cells (RBCs), white blood cells (WBCs), epithelial cells, and casts were observed under a microscope.

#### 2.6.6. Hematology

Approximately 1 mL of the blood taken during the necropsy was placed into a CBC bottle (Vacutainer 3 mL, Becton Dickinson, Franklin Lakes, NJ, USA) containing EDTA-2K anticoagulant. The following parameters were measured with a Coulter counter (ADVIA 2120, Siemens, Ames Division, Miles Laboratory, USA): white blood cell count, red blood cell count, hemoglobin, hematocrit, mean corpuscular volume, mean corpuscular hemoglobin, mean corpuscular hemoglobin concentration, red cell distribution width, hemoglobin distribution width, platelets, mean platelet volume, and WBC differential count (neutrophils, lymphocytes, monocytes, eosinophils, basophils, and large unstained cells). 1.8 mL of the blood taken during the necropsy was dispensed into a microtube containing 0.2 mL 3.2% sodium citrate, and plasma was obtained from centrifugation (Eppendorf, Hamburg, Germany) for 10 min to assess the blood clotting time. The prothrombin time and activated partial thromboplastin time were measured in seconds from plasma using the nephelometric analysis method (Woziwodzki, 1970) with a coagulation time analyzer (ACL 100, Instrumentation Laboratory, Bedford, MA, USA).

#### 2.6.7. Serum Biochemistry

More than 2 mL of the blood taken during the necropsy was added to a 5 mL Vacutainer tube (IMPROBE, Germany) containing a clot activator. The blood was coagulated by maintaining it at room temperature for 15–20 min and was then centrifuged (3,000 rpm, 1,500 RCF, MF300, Hanil, Korea) for 10 min. The following parameters were measured with a serum biochemistry analyzer (AU400, Olympus, Tokyo, Japan): aspartate aminotransferase, alanine aminotransferase, alkaline phosphatase, creatine phosphokinase, total bilirubin, glucose, total cholesterol, triglycerides, total protein, albumin, albumin/globulin ratio, blood urea nitrogen, creatinine, inorganic phosphorus, and calcium ions. The electrolytes were measured with the electrolyte autoanalyzer (644 Na, K, Cl Analyzer, Ciba-Corning, USA).

#### 2.6.8. Necropsy and Organ Weight

Before the scheduled necropsy, all surviving animals were fasted overnight (for 16–20 h) and euthanized with isoflurane (Ifran liquid, Hana Pharm. Co., Korea) inhalation on the day of the necropsy. After anesthesia was confirmed, blood was taken from the posterior vena cava for hematology and serum biochemistry analyses. The abdominal aorta and posterior vena cava were cut to euthanize the animals. All organs of the body surface, subcutis, head, and all internal organs of the abdominal and thoracic cavities were observed grossly. Next, the pituitary gland, ovaries, uterus, adrenal glands, thymus, prostate gland, testes, epididymides, spleen, kidneys, heart, lung, brain, and liver were removed and weighed with an electronic balance (BP221S, Sartorius Co., Gottingen, Lower Saxony, Germany), and all paired organs were measured separately. The absolute organ weights were converted to relative organ weights based on the organ-to-fasted body weight ratios.

#### 2.6.9. Histopathology

Microscopic examinations were performed on the preserved organs and tissues taken from all animals in the vehicle-treated and high dose-treated groups. All gross lesions as defined by the study pathologist were also included in the examination. The eyes and the optic nerves were preserved in Davidson's fixative, and the testes and epididymides were preserved in Bouin's fixative. The following organs and tissues were fixed in 10% neutral buffered formalin solution: the thymus, spleen, pancreas, stomach, duodenum, jejunum, ileum, cecum, colon, rectum, mesenteric lymph nodes, mandibular lymph nodes, salivary gland, thyroid gland (including parathyroid gland), Harderian gland, heart, lung, kidneys, adrenal glands, liver, aorta, brain, pituitary gland, tongue, trachea, esophagus, sternum, thoracic spinal cord, femorotibial joint, peripheral nerve (sciatic), skeletal muscle (femoral), prostate gland, seminal vesicles, ovaries, uterus, vagina, urinary bladder, and skin (including mammary gland).

### 2.7. Statistical Analysis

In the rat studies, data are presented as mean ± standard deviation. Body weight, food and water consumption, total volume of urine, hematology and serum biochemistry, and absolute and relative organ weights were assumed to be normally distributed and were analyzed by a one-way analysis of variance (ANOVA) [24]. The assumption of homogeneity was tested using Levene's test [25]. If the overall ANOVA was significant and the assumption of homogeneity of variance was met, Duncan's multiple-range test was used as a post hoc test to identify significantly different groups from the vehicle control group [26]. Scheffe's test was used if the sample size was unequal between the groups [27]. If the assumption of homogeneity of variance was not met, Dunnett's T3 test was used as the post hoc test [28]. Student's *t*-test was used to analyze the differences in means between the two independent groups. The urinalysis data were rank-transformed and analyzed by the nonparametric Kruskal-Wallis *H*-test [29]. If a statistically significant difference was observed between groups, the Mann-Whitney *U* test was used to identify the groups that were significantly different from the vehicle control group [30]. SPSS 10.1K was used for all statistical analyses (Chicago, IL, USA). A *P* < 0.05 was considered significant.

In the dog studies,data are presented as mean ± standard deviation. Body weight, food and water consumption, total volume of urine, hematology and serum biochemistry, and absolute and relative organ weights were assumed to be normally distributed and were analyzed by a one-way analysis of variance (ANOVA) [24]. SPSS 10.1K was used for all statistical analyses (Chicago, IL, USA). A *P* < 0.05 was considered significant.

## 3. Results

### 3.1. Acute Oral Toxicity Study in Rats

The test substance was orally administered to a single group of five male and five female SD rats at 7 weeks of age in dosage levels of 2 g/mL/kg B.W. Only one female rat given 2 g/mL/kg displayed piloerection and activity decrease 1 day after the treatment and died within the same day. However, the gross necropsy results showed no treatment-related changes and revealed no evidence of specific toxicity related to the test substance. Also, there was a statistically significant decrease in the body weights at 1 day in all test group rats (Table 1). However, the body weights of the test groups recovered. Therefore, this was a temporary side effect from the test substance. But, all rats except the aforementioned female rat were not noted in the mortality, clinical, and gross findings.

### 3.2. Acute Oral Toxicity in Beagle Dogs

#### 3.2.1. Clinical Signs and Mortality

No dogs died during the experimental period. After the administration of the test substance, vomiting was observed. In the male group given 1,000 mg/kg (Table 2), very slight vomiting of the test substance at 3 hr and vomiting of the food at 5 hr were observed in one male. In addition, in the male group given 2,000 mg/kg, severe vomiting was observed in two males at 30 min, and further vomiting of the food was observed in a male on day 5. As shown in Table 3, vomiting of the food was observed in one vehicle-treated female on day 1, moderate vomiting of the test substance was observed in one female given 1,000 mg/kg at 1 hr, and severe vomiting of the test substance was observed in two females given 2,000 mg/kg at 30 min. Vomiting of the food was observed in all female groups on day 7.

#### 3.2.2. Body Weights

The body weight was observed throughout the 15-day experimental period (Table 4). In the males, a loss of body weight was observed in a male given 1,000 mg/kg on days 1 and 7 and in a male given 2,000 mg/kg on day 3. In the females, a loss of the body weight was observed in a vehicle-treated female on day 3, in a female given 1,000 mg/kg on days 1 and 7, and in a female given 2,000 mg/kg on days 1 and 7.

#### 3.2.3. Necropsy

As shown in Table 5, hemorrhage of the mucosa of the duodenum and dark red discoloration in the right lobule of a lung were observed in one male. However, no abnormalities were observed in other males and females.

### 3.3. Subchronic Oral Toxicity Study in Rats

#### 3.3.1. Clinical Signs and Mortality

A rat was found dead on day 50 and another on day 67 after administration in the female group with 1,000 mg/kg/day. In both dead rats, abnormal fur, dirty noses, and hypothermia were observed on the day before death. There were no abnormal findings that were associated with the administration of the test substance other than minor observed signs such as scratched wounds, crust formation, scarring, and the loss of teeth, which occurred at a low frequency with no dose-relationship during the study period (data not shown).

#### 3.3.2. Body Weight and Food and Water Consumption

As a result of the observations of body weight changes and body weight gains for 13 weeks during the administration and observation periods, there were no statistically significant changes in all treatment groups of both sexes compared to the vehicle-treated group. The food consumption in the male group of 360 mg/kg/day was significantly increased at weeks 4 and 10 after the administration compared to the vehicle-treated group (Tables 6 and 7) (*P* < 0.05 or *P* < 0.01). There was no significant change in water consumption in all groups of both sexes compared to the vehicle-treated group.

#### 3.3.3. Ophthalmic Examination

In all groups, there were no particular signs observed from external eye examinations at grouping and ophthalmic examinations using a fundus camera in the final week of administration (data not shown).

#### 3.3.4. Urinalysis

There were no particular signs associated with the administration of the test substance as a result of urinalysis performed in the final administration week (data not shown).

#### 3.3.5. Hematology

The RDW (red cell distribution width) in the male group administered with 1,000 mg/kg/day significantly decreased compared to the vehicle-treated group (Tables 8 and 9) (*P* < 0.01), but no consistent changes were observed in the male or female groups in association with the administration of the test substance in other examination items. There were no significant changes between the groups as a result of measurement of PT (prothrombin time) and APTT (activated partial thromboplastin time) (data not shown).

#### 3.3.6. Serum Biochemistry

As a result of serum biochemical examinations using the serum of collected blood during the necropsy (Tables 10 and 11), dose-related decreasing tendencies of ALT, BUN, GLU, AST, ALP, CHO, PRO, and CPK and dose-related increases of A/G, Na^+^, and Cl^−^ were observed in the males. In addition, statistically significant dose-dependent decreases of ALT, BUN, and GLU were observed compared to the vehicle-treated group (*P* < 0.05 or *P* < 0.01) and significant dose-dependent increases of A/G, Na^+^, and Cl^−^ were observed compared to the vehicle-treated group (*P* < 0.01). In the female groups, no statically significant changes were observed in all measurement items.

#### 3.3.7. Necropsy

As a result of the scheduled necropsy of live animals at 13 weeks after the administration, no notable dose-related changes were observed in either males or females. For males, enlargement of the spleen was observed in one case in the vehicle-treated group and one case in the 600 me/kg/day-treated group, a solid brown nodule of the prostate gland was observed in one case in the vehicle-treated group, and diffuse red spots or a flare-up of the thymus was observed in one case in the vehicle-treated group and one case in the test substance-treated groups. For the females, the retention of a clear fluid in the uterus was observed in the vehicle-treated group and the 360, 600, and 1,000 mg/kg/day-treated groups (2, 4, 3, and 2 cases, resp.) and diffuse red spots in the thymus and dark yellowish brown discoloration of the lung were observed in the 600 mg/kg/day-treated group in two cases and one case, respectively. Additionally, enlargement of the submandibular lymph node was observed in one case in the 1,000 mg/kg/day-treated group. As a result of the necropsy of two dead females of the 1,000 mg/kg/day-treated group, diffuse red spots or dark red coloration in the lung and diffuse red spots in the anterior stomach and in the thymus were observed along with gas retention due to postmortal changes (data not shown).

#### 3.3.8. Organ Weight

For males, the absolute and relative weights of the thymus showed significant increases in the 360 and 600 mg/kg/day-treated groups compared to the vehicle-treated group (Tables 12 and 13) (*P* < 0.05 or *P* < 0.01) and the relative weight of the left epididymis showed a significant decrease in the 600 mg/kg/day-treated group compared to the vehicle-treated group (*P* < 0.05), but these changes were not dose-related. For females, the relative weight of the liver showed a significant increase in the 1,000 mg/kg/day-treated group compared to the vehicle-treated group (*P* < 0.05).

#### 3.3.9. Histopathology

As a result of histopathological examination of rats with a scheduled necropsy, in the males in the vehicle-treated group, severe granulomatous inflammation of the prostate gland, moderate pyelonephritis in the kidney, sight cysts in the pituitary gland, and moderate granuloma in the liver were observed in one case, along with moderate inflammatory cell infiltration in the Hadrian gland and slight inflammatory cell infiltration in the heart (one case each). For the females, two cases of slight tubular regeneration in the kidney were observed in one case each. In the males with a scheduled necropsy from the 1,000 mg/kg/day-treated group, slight tubular remnants in the pituitary gland, slight granuloma in the liver, and a slight dilation of the lumen in the uterus were observed in one case in each group. In the low- and medium-dose groups, pale yellowish brown discoloration of the left lobe of the lungs (identified as focal alveolitis) was observed in one female from the 600 mg/kg/day-treated group, and diffuse red spots in the thymus were observed in two cases (identified as focal hemorrhages). No abnormal findings were observed in the abnormal organs. As a result of the histopathological examinations on the two dead female rats from the 1,000 mg/kg/day-treated group, it was found that the rats had suffered severe postmortal changes, and therefore an accurate evaluation of the lesions was difficult. However, in rats that died on day 67 after the administration, diffuse vacuolation in the adrenal gland, atrophy of the spleen, and inflammatory cell infiltration in the heart were observed to a minor extent, and no abnormal findings were observed in a dead animal on day 50 after the administration.

## 4. Discussion

*Polygala tenuifolia* root is a famous traditional medicine. Although many studies have reported the pharmacological efficacy of the root extract, there is no information on its safety, such as its acute and subchronic oral toxicity. First, we investigated the acute and subchronic oral toxicity of the root extract in rats. The root extract was administered orally at 0 or 2 g/kg body weight for the acute oral toxicity test and at 0, 360, 600, or 1,000 mg/kg body weight for the subchronic oral toxicity test. In the acute oral toxicity study, one female rat given 2 g/kg of the root extract exhibited piloerection and decrease in activity levels 1 day after treatment and died within the same day. The gross findings of the autopsy showed no evidence of specific toxicity related to the test substance. In addition, there was a statistically significant decrease in body weight at day 1 in all test-group rats, but the body weights later recovered. Hence, this finding may show a temporary effect of the test substance. However, all rats except the aforementioned female rat were not noted in the mortality, clinical, and gross findings. These results suggest that the approximate lethal dose for the root extract of* P. tenuifolia* may be ≥2 g/kg in male rats and ≤2 g/kg in female rats under the conditions used in this study. In the subchronic oral toxicity study, there were no abnormal signs associated with administration of the test substance. No specific findings for body weight, consumption of food and water, ophthalmic examination, urinalyses, and blood clotting time were observed in relation to administration of the test substance. In the female group of 1,000 mg/kg/day, rats that died on day 50 and day 67 showed normal clinical signs, body weight changes, and consumption of food and water during the study period but demonstrated abnormal fur, dirty noses or mouths, and hypothermia the day before death. Autopsies revealed dark red coloration or diffuse red spots in the lungs, as well as diffuse red spots in the anterior stomach and thymus gland. A more accurate evaluation of the lesions was made difficult by severe postmortem changes in two rats during the histopathological studies. In the rat that died 67 days after administration of the root extract, diffuse vacuolation in the adrenal glands, atrophy of the spleen, and infiltration of the inflammatory cells in the heart were observed. In the rat that died on day 50, abnormal findings were not observed. As mentioned above, the clinical signs, body weight changes, food and water consumption, necropsy findings, and histopathological findings obtained for the dead rats did not indicate any toxicity of the test substance. Therefore, the deaths of the rats were not associated with the administration of the test substance and were judged to be accidental due to administration errors. This hypothesis is supported by the fact that no abnormal findings were observed in relation to the administration of the test substance in the remaining rats in the same dose group. We demonstrated that the test substance could be used as a food supplement, because some famous food ingredients were toxic at high doses, (e.g., lemongrass (*Cymbopogon citratus*) essential oil,* Roystonea regia* fruit, and *α*-glycerylphosphorylcholine (AGPC)) [31–33]. The results of the serum biochemical examinations of the rat sera revealed reductions in the levels of AST and ALT in the male groups. AST and ALT are coenzymes of pyridoxal phosphate (PALP). PALP is a derivative of vitamin B_6_ and AST and ALT are activated by union with PALP, but nonlinked aminotransferases are not activated. Hence, reductions in the levels of ALT and AST occur if there is low generation of aminotransferases or PALP or if the linkage between the aminotransferases and PALP is disturbed. Cephalosporin-like antibiotics are known to induce reductions in ALT levels, but in toxicity studies, reductions have been mentioned in general and toxicological significance has not been attributed [34, 35]. The blood urea nitrogen (BUN) levels in the blood represent the nitrogen content in relation to urea, which is a metabolite of nitrogen and is closely related to heart diseases. The BUN value might show a decrease, due to a reduction in urea synthesis during toxic hepatitis, severe hepatic impairment, and consuming a low protein diet. However, in the present study, no abnormal findings related to dose were observed. In general, the decrease in BUN level is not a critical factor in toxicity studies [34]. Moreover, these changes were not regarded as adverse effects because they remained within normal ranges [36, 37]. Hematological examinations revealed reductions in red blood cell volume distribution width (RDW) in the male group given 1,000 mg/kg/day. Decreasing tendencies in the levels of GLU, ALP, CHO, PRO, ALB, and CPK and increments in the levels of A/G, Na^+^, and Cl^−^ in male treatment groups were within the normal range [38, 39] and also remained within the normal range when compared with the historical data [21] available at ChemOn Inc. In the vehicle-treated group, increments or reductions in some measurement items were also observed accidentally and were thought to be somewhat contributory to the above results [40]; therefore, it was considered that these changes were toxicological and were not associated with the administration of the test substance. RDW decreased significantly, but the changes remained within the limits of normal biological variations [41, 42]. Significant changes in the absolute/relative weights of the thymus gland in the male groups given 360 and 600 mg/kg/day, the relative weights of the left epididymis in the male groups given 600 mg/kg/day, and the relative weights of the liver in the female group given 1,000 mg/kg/day were not accompanied by abnormal autopsy or histopathological findings. A relationship with sex or dose was not observed, so these changes were not considered to be a toxicological feature of the test substance. Even though the weights of the thymus gland and left epididymis changed significantly, they were within the limits of normal biological variations; this finding was in accordance with results reported by Wang et al. [43]. Regarding relative organ weight, an increase in the liver in the female rats, compared with Banpungtongseong-san reported organ weights analysis, showed that there were statistically significant changes in the absolute weights of the thymus in the male group of 2,000 mg/kg/day. However, these changes were not regarded as adverse effects because they were minor, remained within normal historical control ranges for Fischer 344 rats, and were not correlated with pathological lesions in the respective organs [44, 45]. Autopsy and histopathological examinations revealed abnormal findings in some organs, but the prevalence of these findings was low and these changes were spontaneous and nonspecific [46, 47]. Therefore, the toxicological importance of these findings was negligible. No abnormal findings were observed in association with administration of the test substance upon histopathological examination of abnormal organs in low- and medium-dose groups.

Second, acute oral toxicity of the root extract* P. tenuifolia* in beagle dogs was investigated. The root extract was administered orally at 1,000 or 2,000 mg/kg body weights; the vehicle-treated group was only given gelatin capsules for the single dose oral toxicity test. As a result of the present study, vomiting and inhibition of body weight gain were observed after the administration of the test substance, but other abnormalities in clinical signs, deaths, and abnormal findings in the necropsy results were not observed in relation to administration of the test substance. The results of clinical sign examinations of the dogs observed vomiting in one male given 1,000 mg/kg at 5 hr and in one male given 2,000 mg/kg on day 5. This was after the administration was considered to be unrelated to the test substance because vomiting was also observed in the vehicle-treated female group on day 1, and vomiting like this is commonly observed in dogs which have a well-developed vomiting system. However, these changes were not considered serious when compared with the previous study, as some famous oriental medicine and food compositions were found to be toxic, including* Paecilomyces sinclairii* [48], SH21-B [49], epigallocatechin gallate (EGCG) [50], novel thiazolidinedione (MCC-555) [51], and nelumbinis semen (NS, the seeds of* Nelumbo nucifera*) [52]. Vomiting of the food occurred sporadically in some dogs in all groups, including the controls. This frequency was typical for this colony of dogs [53]. In general, the incidence of vomiting did not suggest an effect of the test substance [54]. Vomiting is a common presenting sign in small animal practice [55]. Moreover, many peripheral stimuli of abdominal structures will initiate vomiting in dogs [56, 57]. The “chemoreceptor trigger zone” of the brainstem has been identified as the area postrema that is located on the dorsal surface of the medulla oblongata adjacent to the caudal end of the fourth ventricle [58]. Therefore, it was not considered to be related to the treatment with the test substance. The loss of body weight was observed in males and females, but because vomiting of the test substance occurred more frequently at 2,000 mg/kg than at 1,000 mg/kg, the absorption of test substance seemed to be relatively higher at 1,000 mg/kg than at 2,000 mg/kg. Also, the loss of body weight showed a temporary serious tendency on days 1 and 3 after the administration. However, the body weights of the male 1,000 mg/kg and 2,000 mg/kg groups recovered. In addition, the vehicle-treated group of females showed a tendency to decrease in body weight from approximately 14 days. In all groups, increment and reduction tendencies were also observed, and these changes were observed [59–62]. The changes in body weights were probably the result of irritation of the alimentary tract [61]. We also considered gastroenteric issues from vomiting as accordingly inducing a disorder of the nutriments absorption [63]. A hemorrhage of the mucosa of the duodenum and the dark discoloration of the lung observed in a male given 2,000 mg/kg were not considered as test substance-related changes, because these were occasionally found in domestic beagle dogs without administration of any of the test substances. Dog experiment findings were generally observed as environmental causes, because these results are ignored [64]. In the necropsy results, a dark red spot on the lung and a light gray color change were observed. However, this change was not dose-related [49], but it was an accidental change. Also, the lungs showed discoloration (dark, mottled, and focus), and the stomach showed discoloration [65, 66]. The gross lesion of the mass or nodule corresponded to the microscopic lesion of the thrombus. These were direct effects of the drug or were secondary to the vascular damage caused by dehydration (with hemodynamic shock) from vomiting and diarrhea [66]. Histopathology changes and hemorrhages in the lung were observed by Werley et al. [67]. However, they were considered to be typical of spontaneously arising background findings, and no other biologically significant effects were observed by histopathology studies on the tissues and organs. The discolorations in the other various organs and tissues were within the range of normal background lesions in dogs of this strain and age and were therefore not considered to be related to the test substance [68].

In this experiment, the administration of the root extract of* Polygala tenuifolia* Willdenow (2 g/kg/day) did not result in acute oral toxicity in the SD rats. The minimum lethal dose (MLD) was also considered to be higher than 1,000 mg/kg, since vomiting of the test substance occurred at 2,000 mg/kg. In the subchronic oral toxicity test, no obvious toxic changes due to administration of the root extract of* P. tenuifolia* were observed in any of the parameters tested (clinical signs, mortality, body weight changes, food and water consumption, ophthalmic examination, urinalyses, hematology, serum biochemistry, organ weights, autopsy, and histopathology). Therefore, under the experimental conditions, the no-observed-adverse-effect level (NOAEL) of the root extract of* P. tenuifolia* was determined to be 1,000 mg/kg/day for both sexes, but the target organ was not established. In conclusion, the present study demonstrated that the acute or subchronic toxicity of the root extract of* Polygala tenuifolia* was not toxic in rats and beagle dogs.

## Figures and Tables

**Table 1:** Body weight values of Sprague-Dawley rats orally treated with *Polygala tenuifolia* extracts in acute oral toxicity study.

Group summary of body weight				
Dosage in days	(Vehicle-treated group, 0 g/10 mL/kg/B.W.)		(Test substance-treated group, 2 g/10 mL/kg/B.W.)	
	Mean ± S.D.	*N*	Mean ± S.D.	*N*
Male				
−1	212.70 ± 2.955	5	240.20 ± 2.953	5
0	193.53 ± 2.767	5	191.71 ± 3.125	5
1	218.14 ± 1.530	5	192.55^*^ ± 13.589	5
7	277.23 ± 8.416	5	255.49^*^ ± 15.501	5
14	328.20 ± 19.827	5	313.22 ± 13.880	5
Female				
−1	197.17 ± 4.587	5	197.16 ± 4.621	5
0	163.89 ± 5.228	5	159.58 ± 4.439	5
1	181.93 ± 8.6178	5	163.81^*^ ± 10.634	4
7	215.62 ± 14.040	5	203.59 ± 11.938	4
14	235.38 ± 21.433	5	231.21 ± 6.933	4

S.D.: Standard deviation.

*N*: Number of animals.

^*^ *P *< 0.05 compared to the vehicle-treated group.

(Unit: g).

**Table 2:** Incidence of clinical signs of male beagle dogs orally treated with *Polygala tenuifolia* extracts in single dose oral toxicity study.

Sex: male				
Day	Signs observed	Group		
		0	1,000	2,000
0-1 (0.5 hours)	Appears normal	2/2^*^	2/2	0/2
	Vomiting (severe)	0/2	0/2	2/2
0–2 (1 hour)	Appears normal	2/2	2/2	2/2
0–3 (2 hours)	Appears normal	2/2	2/2	2/2
0–4 (3 hours)	Appears normal	2/2	1/2	2/2
	Vomiting (mild)	0/2	1/2	0/2
0–5 (4 hour)	Appears normal	2/2	2/2	2/2
0–6 (5 hours)	Appears normal	2/2	1/2	2/2
	Vomiting (food)	0/2	1/2	0/2
0–7 (6 hours)	Appears normal	2/2	2/2	2/2
1	Appears normal	2/2	2/2	2/2
2	Appears normal	2/2	2/2	2/2
3	Appears normal	2/2	2/2	2/2
4	Appears normal	2/2	2/2	2/2
5	Appears normal	2/2	2/2	1/2
	Vomiting (food)	0/2	0/2	1/2
6	Appears normal	2/2	2/2	2/2
7	Appears normal	2/2	2/2	2/2
8	Appears normal	2/2	2/2	2/2
9	Appears normal	2/2	2/2	2/2
10	Appears normal	2/2	2/2	2/2
11	Appears normal	2/2	2/2	2/2
12	Appears normal	2/2	2/2	2/2
13	Appears normal	2/2	2/2	2/2
14	Appears normal	2/2	2/2	2/2

^*^Number of animals with the sign/number of animals examined.

**Table 3:** Incidence of clinical signs of female beagle dogs orally treated with *Polygala tenuifolia* extracts in single dose oral toxicity study.

Sex: female				
Day	Signs observed	Group		
		0	1,000	2,000
0-1 (0.5 hours)	Appears normal	2/2^*^	2/2	0/2
	Vomiting (severe)	0/2	0/2	2/2
0–2 (1 hour)	Appears normal	2/2	1/2	2/2
	Vomiting (moderate)	0/2	1/2	0/2
0–3 (2 hours)	Appears normal	2/2	2/2	2/2
0–4 (3 hours)	Appears normal	2/2	2/2	2/2
0–5 (4 hours)	Appears normal	2/2	2/2	2/2
0–6 (5 hours)	Appears normal	2/2	2/2	2/2
0–7 (6 hours)	Appears normal	2/2	2/2	2/2
1	Appears normal	1/2	2/2	0/2
	Vomiting (food)	1/2	0/2	0/2
2	Appears normal	2/2	2/2	2/2
3	Appears normal	2/2	2/2	2/2
4	Appears normal	2/2	2/2	2/2
5	Appears normal	2/2	2/2	2/2
6	Appears normal	2/2	2/2	2/2
7	Vomiting (food)	2/2	2/2	2/2
8	Appears normal	2/2	2/2	2/2
9	Appears normal	2/2	2/2	2/2
10	Appears normal	2/2	2/2	2/2
11	Appears normal	2/2	2/2	2/2
12	Appears normal	2/2	2/2	2/2
13	Appears normal	2/2	2/2	2/2
14	Appears normal	2/2	2/2	2/2

^*^Number of animals with the sign/number of animals examined.

**Table 4:** Body weight values of beagle dogs orally treated with *Polygala tenuifolia* extracts in single dose oral toxicity study.

Sex: male							
Group	Body weights (kg)						
(mg/kg)	Animal ID	0 d	1 d	3 d	7 d	14 d	Gain
0 (*n* = 2)	1	7.283	7.350	7.694	7.741	8.226	0.943
	2	8.075	8.043	8.097	8.185	8.448	0.373
	Mean	7.679 ± 0.560	7.697 ± 0.490	7.896 ± 0.285	7.963 ± 0.314	8.337 ± 0.157	0.658 ± 0.403

1,000 (*n* = 2)	3	8.785	8.549	8.666	8.551	8.938	0.153
	4	7.961	7.863	8.128	8.068	8.352	0.391
	Mean	8.373 ± 0.583	8.206 ± 0.485	8.397 ± 0.380	8.310 ± 0.342	8.645 ± 0.414	0.272 ± 0.168

2,000 (*n* = 2)	5	8.282	8.225	8.437	8.511	8.918	0.636
	6	7.269	7.595	7.375	7.588	7.959	0.690
	Mean	7.776 ± 0.716	7.910 ± 0.445	7.906 ± 0.751	8.050 ± 0.653	8.439 ± 0.678	0.663 ± 0.038

Sex: female							
Group	Body weights (kg)						
(mg/kg)	Animal ID	0 d	1 d	3 d	7 d	14 d	Gain

0 (*n* = 2)	7	7.145	7.412	7.160	7.308	7.567	0.422
	8	6.531	6.656	6.291	6.875	7.159	0.628
	Mean	6.838 ± 0.434	7.034 ± 0.535	6.726 ± 0.614	7.092 ± 0.306	7.363 ± 0.288	0.525 ± 0.146

1,000 (*n* = 2)	9	7.433	7.465	7.417	7.395	7.307	−0.126
	10	7.046	6.474	6.658	6.660	6.930	−0.116
	Mean	7.240 ± 0.274	6.970 ± 0.701	7.038 ± 0.537	7.028 ± 0.520	7.119 ± 0.267	−0.121 ± 0.007

2,000 (*n* = 2)	11	7.449	7.138	7.238	7.245	7.534	0.085
	12	7.096	6.898	7.111	7.007	7.156	0.060
	Mean	7.273 ± 0.250	7.018 ± 0.170	7.175 ± 0.090	7.126 ± 0.168	7.345 ± 0.267	0.072 ± 0.018

**Table 5:** Gross findings of beagle dogs orally treated with *Polygala tenuifolia* extracts in single dose oral toxicity study.

Sex: male				
Group	Gross observation		Frequency	
(mg/kg)	Location	Gross findings	Death	Survivors
0		No gross findings	^*^0/0	2/2
1,000		No gross findings	0/0	2/2
		No gross findings	0/0	0/2
2,000	Duodenum	Hemorrhage on mucous membrane	0/0	1/2
	Lung	Dark red discoloration of right lobule	0/0	1/2

Sex: female				
Group	Gross observation		Frequency	
(mg/kg)	Location	Gross findings	Death	Survivors

0		No gross findings	^*^0/0	2/2
1,000		No gross findings	0/0	2/2
2,000		No gross findings	0/0	2/2

^*^Number of animals with the sign/Number of animals examined.

**Table 6:** Food consumption of male rats.

Food consumption (g)								
Study: 08-RR-011	Dose (mg/kg/day)				Sex: male			
Weeks	0		360		600		1,000	
	Mean ± S.D.	*N*	Mean ± S.D.	*N*	Mean ± S.D.	*N*	Mean ± S.D.	*N*
0	19.69 ± 0.65	10	19.25 ± 1.81	10	19.90 ± 1.17	10	20.65 ± 1.54	10
1	20.98 ± 1.49	10	20.96 ± 1.26	10	21.20 ± 1.31	10	20.78 ± 1.77	10
2	19.67 ± 1.52	10	21.09 ± 1.51	10	21.00 ± 0.74	10	21.13 ± 1.23	10
3	20.13 ± 1.27	10	21.77 ± 1.06	10	21.70 ± 1.86	10	20.44 ± 1.13	10
4	18.60 ± 0.80	10	20.84 ± 1.30^**^	10	19.65 ± 0.84	10	19.06 ± 0.33	10
5	19.34 ± 0.59	10	20.27 ± 0.80	10	20.26 ± 1.66	10	19.78 ± 1.22	10
6	20.32 ± 0.78	10	20.41 ± 1.18	10	20.15 ± 0.95	10	19.02 ± 1.41	10
7	19.72 ± 0.44	10	19.85 ± 0.66	10	19.77 ± 0.87	10	18.72 ± 1.48	10
8	17.63 ± 0.69	10	18.61 ± 1.56	10	18.35 ± 1.74	10	18.27 ± 1.64	10
9	19.12 ± 1.79	10	19.95 ± 0.99	10	19.05 ± 0.56	10	18.39 ± 0.89	10
10	20.20 ± 1.23	10	21.53 ± 1.01^*^	10	19.90 ± 0.85	10	18.97 ± 0.61	10
11	18.17 ± 0.41	10	19.11 ± 1.44	10	18.40 ± 1.54	10	17.62 ± 0.94	10
12	19.67 ± 1.31	10	20.64 ± 1.35	10	18.85 ± 1.19	10	19.96 ± 2.02	10
13	20.46 ± 1.27	10	21.80 ± 0.77	10	20.61 ± 2.73	10	21.73 ± 0.98	10

^*^ *P* < 0.05 and ^**^ *P* < 0.01 compared to the vehicle-treated group.

**Table 7:** Food consumption of female rats.

Food consumption (g)								
Study: 08-RR-011	Dose (mg/kg/day)				Sex: female			
Weeks	0		360		600		1,000	
	Mean ± S.D.	*N*	Mean ± S.D.	*N*	Mean ± S.D.	*N*	Mean ± S.D.	*N*
0	14.84 ± 0.52	10	14.34 ± 1.59	10	15.23 ± 0.64	10	13.08 ± 2.94	10
1	12.75 ± 1.17	10	13.01 ± 1.33	10	13.46 ± 1.36	10	12.51 ± 0.59	10
2	13.37 ± 0.85	10	12.28 ± 1.35	10	12.14 ± 1.33	10	11.84 ± 2.40	10
3	13.96 ± 0.45	10	13.95 ± 1.41	10	13.91 ± 1.06	10	12.10 ± 3.30	10
4	12.70 ± 1.82	10	12.74 ± 0.64	10	12.37 ± 1.31	10	11.52 ± 1.82	10
5	13.72 ± 1.89	10	14.14 ± 0.71	10	12.96 ± 2.60	10	12.35 ± 1.99	10
6	13.11 ± 1.21	10	13.40 ± 1.55	10	13.59 ± 1.38	10	11.43 ± 1.75	10
7	13.87 ± 0.87	10	12.68 ± 1.13	10	12.16 ± 0.51	10	11.07 ± 3.29	10
8	11.58 ± 1.08	10	12.81 ± 1.23	10	12.68 ± 1.33	10	12.75 ± 1.06	9
9	12.44 ± 1.24	10	13.19 ± 0.42	10	12.65 ± 1.72	10	11.69 ± 1.68	9
10	14.33 ± 1.06	10	13.49 ± 0.81	10	11.81 ± 2.21	10	12.42 ± 1.49	8
11	13.06 ± 1.28	10	12.76 ± 0.70	10	11.64 ± 1.12	10	12.41 ± 1.01	8
12	13.35 ± 0.65	10	13.10 ± 1.30	10	13.33 ± 2.25	10	13.15 ± 0.91	8
13	14.33 ± 1.19	10	14.44 ± 1.43	10	13.68 ± 2.47	10	15.49 ± 1.55	8

**Table 8:** Hematological values (mean ± standard deviation) of male rats in the subchronic oral toxicity study of the root of *Polygala tenuifolia*.

Parameter	Dose (mg/kg/day)			
	0	360	600	1,000
WBC (10^3^/*μ*L)	9.83 ± 2.83	8.19 ± 1.29	8.91 ± 2.14	9.40 ± 1.32
RBC (10^6^/*μ*L)	8.75 ± 0.31	8.81 ± 0.24	8.69 ± 0.33	8.65 ± 0.29
Hemoglobin (g/dL)	15.7 ± 0.5	15.7 ± 0.4	15.4 ± 0.3	15.3 ± 0.5
Hematocrit (%)	46.0 ± 1.4	45.5 ± 1.0	45.0 ± 1.0	44.9 ± 1.5
MCV (fL)	52.5 ± 1.6	51.7 ± 1.3	51.8 ± 1.6	52.0 ± 1.4
MCH (pg)	17.9 ± 0.5	17.8 ± 0.5	17.8 ± 0.4	17.7 ± 0.4
MCHC (g/*μ*L)	34.1 ± 0.3	34.5 ± 0.4	34.4 ± 0.3	34.1 ± 0.5
RDW (%)	12.0 ± 0.3	11.8 ± 0.3	11.8 ± 0.2	11.6 ± 0.3^**^
HDW (g/dL)	2.35 ± 0.05	2.35 ± 0.08	2.35 ± 0.09	2.32 ± 0.12
Platelet (10^3^/*μ*L)	1061.1 ± 106.5	1030.4 ± 59.9	1043.8 ± 38.4	1103.0 ± 64.7
MPV (fL)	7.0 ± 0.7	6.8 ± 0.7	6.8 ± 0.7	7.0 ± 0.7
Reticulocyte (%)	2.00 ± 0.28	1.84 ± 0.31	1.90 ± 0.31	1.83 ± 0.18

*N*	10	10	10	10

WBC: white blood cell count; RBC: red blood cell count; MCV: mean corpuscular volume; MCH: mean corpuscular hemoglobin concentration; MCHC; mean corpuscular hemoglobin concentration; RDW: red cell distribution width; HDW: hemoglobin distribution width; MPV: mean platelet volume.

^**^Significant difference at *P* < 0.01 compared with the vehicle-treated group.

**Table 9:** Hematological values (mean ± standard deviation) of female rats in the subchronic oral toxicity study of the root of *Polygala tenuifolia*.

Parameter	Dose (mg/kg/day)			
	0	360	600	1,000
WBC (10^3^/*μ*L)	4.60 ± 0.63	4.75 ± 0.94	5.52 ± 1.92	5.11 ± 0.79
RBC (10^6^/*μ*L)	7.89 ± 0.23	7.98 ± 0.36	7.87 ± 0.38	7.78 ± 0.16
Hemoglobin (g/dL)	14.3 ± 0.6	14.7 ± 0.7	14.1 ± 0.6	14.6 ± 0.3
Hematocrit (%)	41.2 ± 1.3	42.1 ± 2.5	40.9 ± 1.9	41.9 ± 0.8
MCV (fL)	52.3 ± 2.0	52.8 ± 1.1	52.0 ± 1.6	53.9 ± 1.1
MCH (pg)	18.2 ± 0.9	18.5 ± 0.3	18.0 ± 0.7	18.8 ± 0.6
MCHC (g/*μ*L)	34.7 ± 0.7	35.0 ± 0.4	34.6 ± 0.5	34.9 ± 0.5
RDW (%)	10.8 ± 0.4	10.6 ± 0.3	10.8 ± 0.4	10.6 ± 0.3
HDW (g/dL)	2.28 ± 0.19	2.22 ± 0.13	2.21 ± 0.16	2.33 ± 0.15
Platelet (10^3^/*μ*L)	1104.4 ± 103.0	1166.6 ± 89.7	1194.1 ± 166.4	1134.0 ± 88.3
MPV (fL)	6.9 ± 0.9	6.5 ± 0.44	7.0 ± 0.9	6.5 ± 0.6
Reticulocyte (%)	2.05 ± 0.60	1.91 ± 0.20	1.97 ± 0.32	2.08 ± 0.46

*N*	9	10	10	8

WBC: white blood cell count; RBC: red blood cell count; MCV: mean corpuscular volume; MCH: mean corpuscular hemoglobin concentration; MCHC: mean corpuscular hemoglobin concentration; RDW: red cell distribution width; HDW: hemoglobin distribution width; MPV: mean platelet volume.

**Table 10:** Serum biochemical values (mean ± standard deviation) of male rats in the subchronic oral dose toxicity study of the root of *Polygala tenuifolia*.

Parameter	Dose (mg/kg/day)			
	0	360	600	1,000
Number of animals examined	10	10	10	10

AST (IU/L)	105.0 ± 26.6	93.4 ± 24.6	87.9 ± 18.5	80.5 ± 11.7
ALT (IU/L)	60.1 ± 12.7	46.6 ± 7.5	44.6 ± 6.8^*^	40.0 ± 5.0^**^
ALP (IU/L)	121.2 ± 32.8	104.4 ± 29.7	105.1 ± 15.2	99.5 ± 17.5
BUN (mg/dL)	18.9 ± 3.5	15.0 ± 1.8^*^	14.6 ± 1.4^*^	14.3 ± 1.6^*^
CRE (mg/dL)	0.64 ± 0.09	0.56 ± 0.03	0.54 ± 0.04	0.60 ± 0.06
GLU (mg/dL)	139.8 ± 18.1	122.9 ± 11.8^**^	125.9 ± 11.1^*^	124.1 ± 8.8^*^
CHO (mg/dL)	127.2 ± 38.6	107.4 ± 12.3	95.2 ± 11.7	98.0 ± 11.1
PRO (g/dL)	7.51 ± 1.12	6.56 ± 0.18	6.50 ± 0.26	6.4 ± 0.16
CPK (IU/L)	195.4 ± 127.9	141.4 ± 96.3	98.2 ± 78.8	93.3 ± 68.6
ALB (g/dL)	3.51 ± 0.43	3.25 ± 0.08	3.24 ± 0.12	3.24 ± 0.09
BIL (mg/dL)	0.22 ± 0.02	0.20 ± 0.02	0.20 ± 0.02	0.20 ± 0.01
TG (mg/dL)	60.8 ± 16.8	57.2 ± 9.8	53.7 ± 6.7	63.2 ± 15.0
IP (mg/dL)	8.42 ± 1.56	6.92 ± 0.21	7.18 ± 0.63	7.49 ± 0.34
Ca^2+^ (mg/dL)	11.81 ± 1.75	10.11 ± 0.15	10.18 ± 0.23	10.13 ± 0.28
A/G (ratio)	0.89 ± 0.07	0.99 ± 0.03^**^	1.00 ± 0.04^**^	1.02 ± 0.04^**^
Na^+^ (mmol/L)	140.5 ± 4.22	144.5 ± 1.08^**^	144.4 ± 1.07^**^	143.9 ± 0.74^**^
K^+^ (mmol/L)	4.39 ± 0.15	4.47 ± 0.27	4.50 ± 0.25	4.49 ± 0.15
Cl^−^ (mmol/L)	104.2 ± 2.97	107.4 ± 0.84^**^	107.6 ± 0.7^**^	107.3 ± 0.82^**^

*N*	10	10	10	10

^∗/∗∗^Significant difference at *P* < 0.05/0.01 compared with the vehicle-treated group.

**Table 11:** Serum biochemical values (mean ± standard deviation) of female rats in the subchronic oral dose toxicity study of the root of *Polygala tenuifolia*.

Parameter	Dose (mg/kg/day)			
	0	360	600	1,000
Number of animals examined	10	10	10	10

AST (IU/L)	91.1 ± 14.0	94.0 ± 17.8	85.9 ± 16.7	86.8 ± 14.0
ALT (IU/L)	35.5 ± 5.8	34.5 ± 4.1	32.9 ± 5.1	32.6 ± 5.3
ALP (IU/L)	64.1 ± 14.9	74.8 ± 9.9	73.8 ± 20.8	71.2 ± 10.1
BUN (mg/dL)	19.3 ± 3.0	17.2 ± 1.9	17.4 ± 1.9	18.4 ± 1.6
CRE (mg/dL)	0.65 ± 0.07	0.64 ± 0.06	0.66 ± 0.11	0.67 ± 0.09
GLU (mg/dL)	116.2 ± 12.7	119.2 ± 11.6	111.7 ± 7.3	110.4 ± 11.0
CHO (mg/dL)	97.2 ± 17.7	106.3 ± 16.7	112.9 ± 19.4	120.4 ± 15.1
PRO (g/dL)	6.23 ± 0.15	6.36 ± 0.29	6.23 ± 0.32	6.11 ± 0.19
CPK (IU/L)	134.8 ± 60.9	120.2 ± 84.3	115.9 ± 82.1	121.9 ± 63.4
ALB (g/dL)	3.23 ± 0.09	3.31 ± 0.15	3.26 ± 0.15	3.20 ± 0.12
BIL (mg/dL)	0.20 ± 0.02	0.21 ± 0.02	0.21 ± 0.03	0.21 ± 0.02
TG (mg/dL)	43.9 ± 5.2	42.9 ± 7.5	52.8 ± 16.3	47.8 ± 8.3
IP (mg/dL)	6.00 ± 0.85	6.15 ± 1.22	6.42 ± 0.99	6.46 ± 0.45
Ca^2+^ (mg/dL)	9.61 ± 0.13	9.78 ± 0.46	9.71 ± 0.17	9.63 ± 0.15
A/G (ratio)	1.08 ± 0.05	1.09 ± 0.08	1.10 ± 0.07	1.11 ± 0.08
Na^+^ (mmol/L)	143.3 ± 1.06	143.6 ± 0.97	142.7 ± 1.06	142.5 ± 1.20
K^+^ (mmol/L)	4.24 ± 0.19	4.57 ± 1.25	4.15 ± 0.27	4.24 ± 0.18
Cl^−^ (mmol/L)	108.3 ± 1.16	109.1 ± 1.20	109.6 ± 0.84	109.88 ± 1.64

*N*	10	10	10	8

**Table 12:** Absolute and relative organ weight of males in the subchronic oral dose toxicity study of the root of *Polygala tenuifolia*.

Parameter	Dose (mg/kg/day)			
	0	360	600	1,000
Body weight	444.51 ± 22.61	460.88 ± 39.59	450.34 ± 30.11	437.99 ± 19.81

Adrenal gland, left	0.0304 ± 0.0048	0.0282 ± 0.0039	0.0295 ± 0.0038	0.0300 ± 0.0045
Per body weight (%)	0.0069 ± 0.0011	0.0061 ± 0.0007	0.0065 ± 0.0006	0.0068 ± 0.0010
Adrenal gland, right	0.0292 ± 0.0045	0.0285 ± 0.0039	0.0288 ± 0.0035	0.0284 ± 0.0042
Per body weight (%)	0.0066 ± 0.0011	0.0062 ± 0.0009	0.0064 ± 0.0006	0.0065 ± 0.0009
Pituitary gland	0.0143 ± 0.0031	0.0131 ± 0.0013	0.0126 ± 0.0018	0.0130 ± 0.0032
Per body weight (%)	0.0032 ± 0.0006	0.0028 ± 0.0002	0.0028 ± 0.0003	0.0030 ± 0.0007
Thymus	0.2515 ± 0.0423	0.3159 ± 0.0400^**^	0.3294 ± 0.0524^**^	0.2891 ± 0.0583
Per body weight (%)	0.0570 ± 0.0115	0.0686 ± 0.0067^*^	0.0734 ± 0.0120^**^	0.0660 ± 0.0132
Prostate	0.5877 ± 0.1284	0.5855 ± 0.1785	0.5839 ± 0.1195	0.6297 ± 0.1285
Per body weight (%)	0.1330 ± 0.0325	0.1278 ± 0.0404	0.1289 ± 0.0196	0.1436 ± 0.0280
Testis, left	2.9152 ± 0.1365	2.0512 ± 0.1353	2.0208 ± 0.1221	2.0452 ± 0.1618
Per body weight (%)	0.4546 ± 0.0383	0.4473 ± 0.0410	0.4495 ± 0.0245	0.4667 ± 0.0251
Testis, left	2.0112 ± 0.1409	2.0550 ± 0.1629	2.0063 ± 0.1262	2.0337 ± 0.1905
Per body weight (%)	0.4539 ± 0.0414	0.4484 ± 0.0486	0.4464 ± 0.0278	0.4636 ± 0.0269
Epididymis, left	0.6852 ± 0.0348	0.6768 ± 0.0397	0.6403 ± 0.0492	0.6961 ± 0.0583
Per body weight (%)	0.1546 ± 0.0108	0.1474 ± 0.0093	0.1423 ± 0.0085	0.1590 ± 0.0119
Epididymis, right	0.7013 ± 0.0830	0.6667 ± 0.0476	0.6459 ± 0.0381	0.6834 ± 0.0461
Per body weight (%)	0.1580 ± 0.0177	0.1452 ± 0.0113	0.1436 ± 0.0057	0.1561 ± 0.0103
Spleen	0.8944 ± 0.2307	0.8399 ± 0.0698	0.8576 ± 0.1300	0.8627 ± 0.0864
Per body weight (%)	0.2019 ± 0.0562	0.1828 ± 0.0154	0.1899 ± 0.0215	0.1969 ± 0.0173
Kidney, left	1.3475 ± 0.1109	1.3537 ± 0.1786	1.3185 ± 0.1704	1.2721 ± 0.1128
Per body weight (%)	0.3032 ± 0.0174	0.2930 ± 0.0189	0.2923 ± 0.0259	0.2903 ± 0.0190
Kidney, right	1.3439 ± 0.1324	1.3803 ± 0.1793	1.3786 ± 0.1808	1.2969 ± 0.1078
Per body weight (%)	0.3020 ± 0.0167	0.2989 ± 0.0216	0.3057 ± 0.0274	0.2960 ± 0.0180
Heart	1,3823 ± 0.1325	1.5006 ± 0.1825	1.5012 ± 0.1357	1.4132 ± 0.1456
Per body weight (%)	0.3017 ± 0.0197	0.3252 ± 0.0241	0.3333 ± 0.0200	0.3225 ± 0.0271
Lung	1.9149 ± 0.1630	1.8571 ± 0.1483	1.8949 ± 0.1652	1.8769 ± 0.1039
Per body weight (%)	0.4314 ± 0.0363	0.4043 ± 0.0327	0.4211 ± 0.0302	0.4288 ± 0.0210
Brain	2.0127 ± 0.0602	1.9798 ± 0.0874	2.0014 ± 0.0659	1.9522 ± 0.0843
Per body weight (%)	0.4539 ± 0.0237	0.4313 ± 0.0254	0.4457 ± 0.0248	0.4459 ± 0.0135
Liver	11.7210 ± 1.0504	11.5963 ± 1.1787	11.4193 ± 1.1750	11.2844 ± 0.0850
Per body weight (%)	2.6348 ± 0.1369	2.5149 ± 0.1118	2.5319 ± 0.1342	2.5752 ± 0.1174

*N*	10	10	10	10

Body weight before necropsy and after fasting.

^∗/∗∗^Significant difference at *P *< 0.05/0.01 compared with the vehicle-treated group.

**Table 13:** Absolute and relative organ weight of females in the subchronic oral dose toxicity study of the root of *Polygala  tenuifolia*.

Parameter	Dose (mg/kg/day)			
	0	360	600	1,000
Body weight	260.11 ± 15.83	259.59 ± 6.80	253.95 ± 17.41	250.61 ± 7.87

Ovary, left (g)	0.0505 ± 0.0119	0.0547 ± 0.0081	0.0467 ± 0.0138	0.0537 ± 0.0067
Per body weight (%)	0.0193 ± 0.0040	0.0211 ± 0.0031	0.0183 ± 0.0045	0.0214 ± 0.0025
Ovary, right	0.0532 ± 0.0072	0.0526 ± 0.0064	0.0515 ± 0.0092	0.0538 ± 0.0058
Per body weight (%)	0.0204 ± 0.0025	0.0203 ± 0.0026	0.0203 ± 0.0032	0.0215 ± 0.0024
Adrenal gland, left	0.0332 ± 0.0073	0.0339 ± 0.0030	0.0324 ± 0.0052	0.0370 ± 0.0041
Per body weight (%)	0.0128 ± 0.0025	0.0131 ± 0.0010	0.0128 ± 0.0020	0.0148 ± 0.0019
Adrenal gland, right	0.0279 ± 0.0044	0.0309 ± 0.0046	0.0301 ± 0.0036	0.0319 ± 0.0068
Per body weight (%)	0.0107 ± 0.0016	0.0119 ± 0.0017	0.0119 ± 0.30015	0.0128 ± 0.0029
Pituitary gland	0.0148 ± 0.0053	0.0137 ± 0.0011	0.0134 ± 0.0015	0.0141 ± 0.0019
Per body weight (%)	0.0056 ± 0.0016	0.0053 ± 0.0004	0.0053 ± 0.0004	0.0056 ± 0.0009
Thymus	0.2235 ± 0.0323	0.2292 ± 0.0349	0.2243 ± 0.0232	0.2290 ± 0.0320
Per body weight (%)	0.0863 ± 0.0142	0.0882 ± 0.0127	0.0883 ± 0.0068	0.0915 ± 0.0128
Uterus	0.6040 ± 0.1695	0.5195 ± 0.1438	0.4848 ± 0.1573	0.6109 ± 0.3313
Per body weight (%)	0.2347 ± 0.0730	0.2002 ± 0.0548	0.1929 ± 0.0678	0.2436 ± 0.1313
Spleen	0.5954 ± 0.596	0.5786 ± 0.0512	0.5402 ± 0.1754	0.6280 ± 0.3671
Per body weight (%)	0.2287 ± 0.0147	0.2228 ± 0.0174	0.2111 ± 0.0673	0.2506 ± 0.1340
Kidney, left	0.7683 ± 0.0657	0.7544 ± 0.0510	0.7713 ± 0.1081	0.7513 ± 0.4212
Per body weight (%)	0.2953 ± 0.0162	0.2905 ± 0.0164	0.3049 ± 0.0495	0.3000 ± 0.1856
Kidney, right	0.7547 ± 0.0548	0.7553 ± 0.0511	0.7900 ± 0.1248	0.7618 ± 0.4748
Per body weight (%)	0.2898 ± 0.0117	0.2909 ± 0.0178	0.3124 ± 0.0572	0.3041 ± 0.1993
Heart	0.9126 ± 0.0678	0.8911 ± 0.0619	0.9117 ± 0.1012	0.9030 ± 0.6912
Per body weight (%)	0.3511 ± 0.0197	0.3433 ± 0.0225	0.3588 ± 0.0280	0.3607 ± 0.3053
Lung	1.4077 ± 0.0881	1.4371 ± 0.0983	1.4229 ± 0.2161	1.4301 ± 0.9812
Per body weight (%)	0.5418 ± 0.0275	0.5535 ± 0.0329	0.5628 ± 0.1006	0.5704 ± 0.3008
Brain	1.8129 ± 0.0649	1.8308 ± 0.0685	1.8026 ± 0.0750	1.8440 ± 0.5932
Per body weight (%)	0.6982 ± 0.0274	0.7058 ± 0.0358	0.7123 ± 0.0485	0.7367 ± 0.3932
Liver	6.0596 ± 0.2314	6.3790 ± 0.6585	6.4577 ± 0.5182	6.4618 ± 0.4515
Per body weight (%)	2.3339 ± 0.0996	2.4553 ± 0.2191	2.5459 ± 0.1755	2.5778 ± 0.1469^*^

*N*	10	10	10	8

Body weight before necropsy and after fasting.

^*^Significant difference at *P* < 0.05 compared with the vehicle-treated group.
